# Supplementary figures and images for: Systems Modeling Reveals Shared Metabolic Dysregulation and Potential Treatments in ME/CFS and Long COVID
Source: Int J Mol Sci. 2025 Jun 25;26(13):6082. doi: 10.3390/ijms26136082 (PMC12250530; doi:10.3390/ijms26136082)

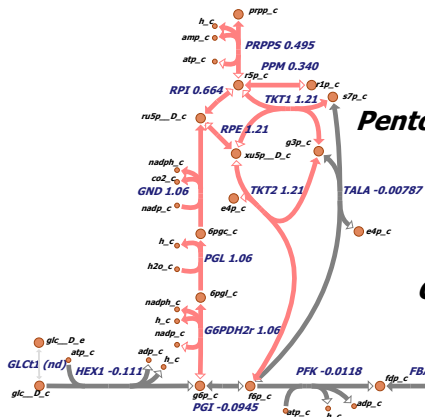

## Pentose Phosphate Pathway

## Glycolysis

## Urea cycle

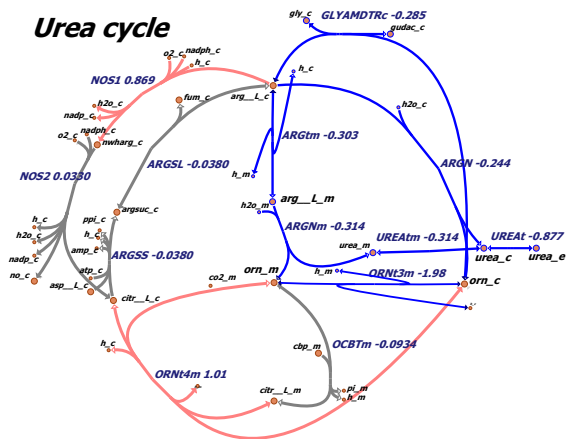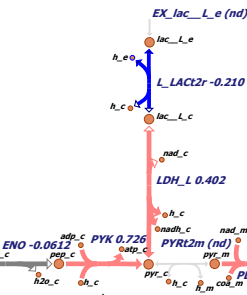

## TCA Cycle

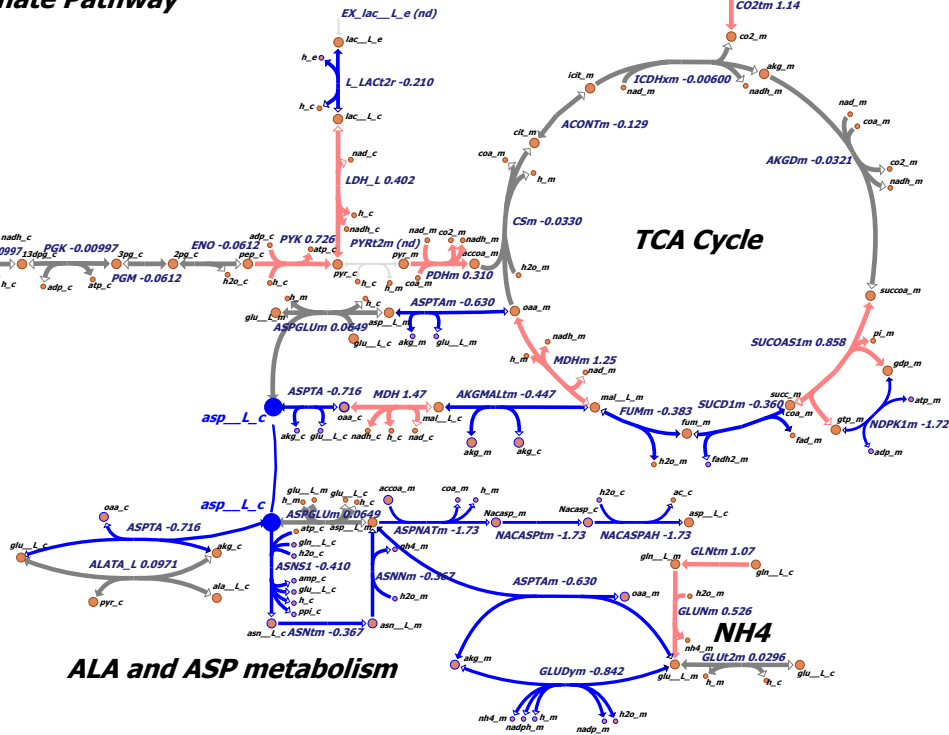

## ALA and ASP metabolism

Supplement: Supplementary file 1 [file ijms-26-06082-s001.zip › Supplymentary_Figure 1.pdf]
